# Serious Game for the Screening of Central Auditory Processing Disorder in School-Age Children: Development and Validation Study

**DOI:** 10.2196/40284

**Published:** 2023-04-26

**Authors:** Ana-Marta Gabaldón-Pérez, María Dolón-Poza, Martina Eckert, Nuria Máximo-Bocanegra, María-Luisa Martín-Ruiz, Iván Pau De La Cruz

**Affiliations:** 1 Grupo de Investigación Innovación Tecnológica para las Personas (InnoTep) Departamento de Ingeniería Telemática y Electrónica ETSIS de Telecomunicación, Campus Sur, Universidad Politécnica de Madrid Madrid Spain; 2 Centro de Investigación en Tecnologías Software y Sistemas Multimedia para la Sostenibilidad (CITSEM) Universidad Politécnica de Madrid Madrid Spain; 3 Department of Physiotherapy, Occupational Therapy, Rehabilitation, and Physical Medicine Faculty of Health Sciences, Universidad Rey Juan Carlos Madrid Spain

**Keywords:** serious games, central auditory processing disorder, process mining, screening, school environment, children

## Abstract

**Background:**

Currently, many central auditory processing disorder screening tests are available for children, and serious games (SGs) are frequently used as a tool for the diagnosis of different neural deficits and disorders in health care. However, it has not been possible to find a proposal that unifies both ideas. In addition, the validation and improvement of SGs, in general, does not take into account the player-game interaction, thus omitting valuable information about the playability and usability of the game.

**Objective:**

This study presented Amalia’s Planet, a game conceived for use in school environments, which allows a first assessment of a child through their performance of the proposed tasks related to different aspects of auditory performance. In addition, the game defines a series of events in relation to the execution of the tasks, which were evaluated for the subsequent optimization of its performance and the improvement of its usability.

**Methods:**

Using screening tools based on the use of SG technologies, a total of 87 school-age children were evaluated to test the various hypotheses proposed in this study. By grouping users according to whether they had personal history of hearing pathologies, the discriminant power, playability, and usability of the final solution were examined using traditional statistical techniques and process mining (PM) algorithms.

**Results:**

With a confidence level of 80% for test 2 (*P*=.19), there was no statistical evidence to reject the null hypothesis that a player’s performance is affected by whether the player had a previous auditory pathology. Furthermore, the tool allowed the screening of 2 players initially categorized as *healthy* because of their low level of performance in the tests and the similarity of their behavior with that of the group of children with a previous pathology. With regard to the validation of the proposed solution, the use of PM techniques made it possible to detect the existence of events that lasted too long, which can lead to player frustration, and to discover small structural flaws in the game.

**Conclusions:**

SGs seem to be an appropriate tool for the screening of children at risk of central auditory processing disorder. Moreover, the set of PM techniques provides a reliable source of information regarding the playability and usability of the solution to the development team, allowing its continuous optimization**.**

## Introduction

### Goal of This Study

Owing to their high degree of neuroplasticity, children and young people can be highly influenced by the stimuli they receive from the environment around them. Given the nature of central auditory processing disorder (CAPD), it is believed that the inclusion of auditory stimulation may lead to functional changes in the individual’s central auditory nervous system [1]. More specifically, “children come to experience greater and more complex demands on central auditory processing as they face more academically, intellectually and linguistically demanding challenges” [2].

Before the therapeutic treatment of CAPD, it is necessary to carry out a diagnostic or screening process so that the presence of the pathology can be detected, thus facilitating a prognosis and the suppression or reduction of the limitations that it may cause in the patient. Among the diagnostic and rehabilitation tools for different pathologies are the well-known serious games (SGs). According to Piaget [3], “games form a part of the child’s intelligence, because they represent the functional or reproductive assimilation of the reality.” However, despite the existence of numerous screening tests for CAPD [4] and the more than palpable efficacy of SGs in multiple settings [5], it has not been possible to find a project in which these 2 techniques are unified to support the screening of individuals who may have the disorder in question.

In the field of SGs, there is a wide variety of methodologies that guide the development and validation of such tools according to their purpose [6-8]. Despite this, most game evaluation and optimization processes focus on game performance, omitting a complete analysis of the player-game interaction, which includes other relevant aspects such as playability, usability, and game understanding. At this point, it is important to highlight the fact that the term *usability* refers to “the extent to which a system (product or service) can be used to achieve the goals with effectiveness, efficiency, and satisfaction in a specified use context” [9].

Process mining (PM) techniques can be used to deepen our knowledge about this player-game interaction. PM allows for the discovery of patterns and tendencies, even without previous models, enabling and improving the comprehension of real processes by recording events. “Process Mining aims to discover, monitor, and improve real processes by extracting knowledge from event logs readily available in today’s information systems” [10].

In view of this, the main objective of this research was to develop and validate a screening tool for CAPD based on the SGs technology, supporting the diagnosis of the disorder in school-age children. Amalia’s Planet was conceived as a game for use in school environments, which allows a first assessment of a child through their achievement of the different proposed tasks related to auditory discrimination, lateralization and localization of sounds, and different aspects of auditory performance. In addition, the game defines a series of events in relation to the execution of the tasks, which were evaluated using PM techniques for the subsequent optimization of its performance and improvement of its usability [11].

It is important to note that throughout this paper, SG is defined according to the study by Tarja et al [12], which stated that an SG is “any game whose main purpose goes beyond the mere entertainment.”

### Background

The American Speech-Language-Hearing Association uses the term CAPD to refer to deficits in the neural processing of auditory information in the central auditory nervous system not due to higher-order language or cognition, as demonstrated by poor performance in one or more of the skills used to preserve, refine, analyze, modify, organize, and interpret information from the auditory periphery [13].

As indicated in the technical reports published by the Working Group on Auditory Processing Disorders, the process of training the abilities affected by CAPD should be implemented as early as possible, taking advantage of the plasticity and cortical reorganization characteristics of the human brain [13,14]. This principle of action could have a much more positive impact on children because the maturation of the cerebral cortex and creation of neural interconnections occur during childhood. Therefore, from the perspective of the consequent therapeutic process, diagnostic testing or screening for CAPD in school-age children is highly desirable. Undiagnosed children often present with learning disabilities, occasionally mistaken for other pathologies simply because auditory processing problems were not considered.

Currently, there are different tools that allow the observation and subsequent screening and diagnosis of CAPD in individuals. Focusing on children, the Hearing in Noise Test for Children [15] lists a total of 130 sentences to determine the quality of information received by an individual in a noise context. Similarly, the Pediatric Speech Intelligibility test proposes a context of overlapping sounds, with relevant and distracting information being uttered in the same or opposite ears [16,17]. The Test Everyday Attention for Children [18] focuses on the assessment of different aspects of an individual that determine their attention levels [19]. Despite their proven efficacy, all these tests tend to be time-consuming and depend on a high degree of specialization on the part of professionals, which restricts their accessibility for the general child population.

Through the use of SGs, a greater number of children can simultaneously take different screening tests, requiring only the company of their relatives or educators and being able to carry out the activity in a familiar environment. Added to these facilities is the fact that because evaluation is performed using a game, the child is stimulated during the process. Finally, another notable advantage of the use of these technologies is that they allow for their evaluation and consequent evolution, making it possible to add different criteria or informative factors in the future as well as to optimize the existing ones. Despite these advantages and the existence of some games focused on the therapeutic process of CAPD, it has not been possible to find a game with these characteristics for the screening of children with CAPD [20-23].

The self-evaluation feature and potential evolution of the games are made possible by the validation process carried out after the use of the games by the end users. During this process, the characteristics of the tool, such as its playability, usability, and discriminating power, must be evaluated. Owing to the lack of publications related to the generation of CAPD-screening SGs for children, different methodologies proposed for the development and validation of this type of technology in the health field have been reviewed. However, most of them base the validation of the tool on performance indicators without assessing aspects of the individual or the tool that may alter the value of these parameters. In the first place, Verschueren et al [24] generated a framework for the conception of SGs for health. This framework was created to ensure that the final product is relevant, theoretically driven, and evidence based through 5 stages. More centered on auditory impairments, Cano et al [25] carried out an exhaustive study of different methodologies for the conception of SGs. According to the authors, all methodologies lacked a register of the player’s learning in a quantitative manner. After learning about the successes and failures of these methodologies, they created the *metodología para la concepción de juegos serios para niños con discapacidad auditiva* (MECONESIS) methodology. MECONESIS is a methodology consisting of 4 stages, namely analysis, preproduction, production, postproduction, and is based on 6 models, namely the analysis, user, pedagogical objective, task, scenario, and validation models.

Among the methodologies that take into account the player-game interaction, the one proposed by Serrano et al [26] in 2014 stands out. The authors proposed a “framework to improve evaluation in educational games.” On the basis of learning analytics and game analytics techniques, the authors suggest a Learning Analytic Model and Learning Analytic System, in which the authors point to some events carried out by players on a graphical interface, the evolution of game stages, and even some logic events. Despite all these sources of information, no tool or model capable of analyzing the collected information was provided.

PM, as a set of techniques designed to extract information from existing data in activity log files, draws on the so-called *event logs* or *game events* from different systems, allows them to be cleaned, establishes contextual relationships, and presents them graphically to facilitate decision-making [27]. This tool provides great advantages to those who use it, among which 3 are worth highlighting in this study [9,28]:

1. PM is based on real events; therefore, the information collected and analyzed is a reflection of reality. It is obvious that, in general, when extracting information for reaching a series of conclusions, the aim is to gather information that is as representative of reality as possible. However, traditional statistical techniques, for example, try to extract such information from a sample of a specific population (sampling techniques). That is, a sampling behavior is generalized to the entire population. In addition, statistics have techniques for the elimination of so-called missing values and outliers so that their presence does not affect the final conclusions that can be reached. For its part, PM works with all the information collected in the *event logs*, thanks to its high degree of automation. Thus, behaviors are neither generalized nor modified. Atypical behaviors are taken into account and compared with standard or normal behaviors.
2. It includes a set of defined algorithms, which allow complex events to be analyzed and conclusions to be drawn to improve the system. That is, PM provides the capability to analyze and optimize the system. Every system, tool, and environment in its first phase of development and throughout its use must be evaluated and updated for its correct functioning. The aim is not only to optimize the system in terms of performance but also to adapt the system to the end user so that it provides the desired usability.
3. It presents the analyzed information in a visual manner, making it easily understandable by any user, to facilitate the decision-making process.

All these advantages make PM the ideal tool for the future optimization and improvement of the SG proposed in this study.

## Methods

### Overview

Amalia’s Planet was conceived as an SG for use in the school environment, which allows a first detection of children at risk of CAPD through their completion of the different tasks proposed. Therefore, through the game and the proposed activities, schools will be able to perform screening in cases in which there is no suspicion of CAPD or in which such suspicion is not entirely clear. In addition, the game defines a series of events in relation to the execution of the tasks, which were evaluated using PM techniques for the subsequent optimization of its performance and improvement of its usability.

From the aforementioned statement, 2 conclusions regarding the approach to the solution are evident. First, a multidisciplinary team must be assembled to cover the wide variety of fields of expertise required for the correct deployment of the solution. In this regard, the team was composed of 3 engineers; 1 PM expert; 1 occupational therapist; 2 audiologists; and 1 ear, nose, and throat specialist. Second, the development of the solution must be approached from 2 points of view: in terms of the health field, there is a need to develop a screening tool with discriminant power for children at risk of CAPD and, in terms of new technologies and data analysis, there is a need to develop of a game that provides relevant information related to different aspects of playability and usability to allow for its evaluation and subsequent optimization.

Nonetheless, although the existence of the aforementioned 2 approaches (health and technological or analytical) is clear, it should not be forgotten that the proposed solution is one and has to cover all the requirements that have been imposed in each of the 2 approaches. This is why, later in this paper, both approaches are mentioned simultaneously.

### Ethics Approval

This study was approved by the Ethics Committee of the Rey Juan Carlos University (reference 1203201805218). The trial was conducted in accordance with the Declaration of Helsinki, as amended in October 2013 by the 64th World Medical Association General Assembly [29].

### Amalia’s Planet for Screening Children at Risk of CAPD

As there is no unified screening method for CAPD, current standard screening methods for CAPD include observing the listening process, recording the patient’s behavior, and performing a series of tests that examine the patient’s auditory function performance [30]. In general, individuals with CAPD have difficulty discriminating sounds, impaired ability to determine the stimulated ear and location of the sound source, and problems processing nonverbal acoustic signals and recognizing the order or pattern of presentation of certain stimuli [31]. These and other conditions experienced by affected individuals should serve as indicators for the tool so that it can detect the presence of the pathology.

**Figure 1:**
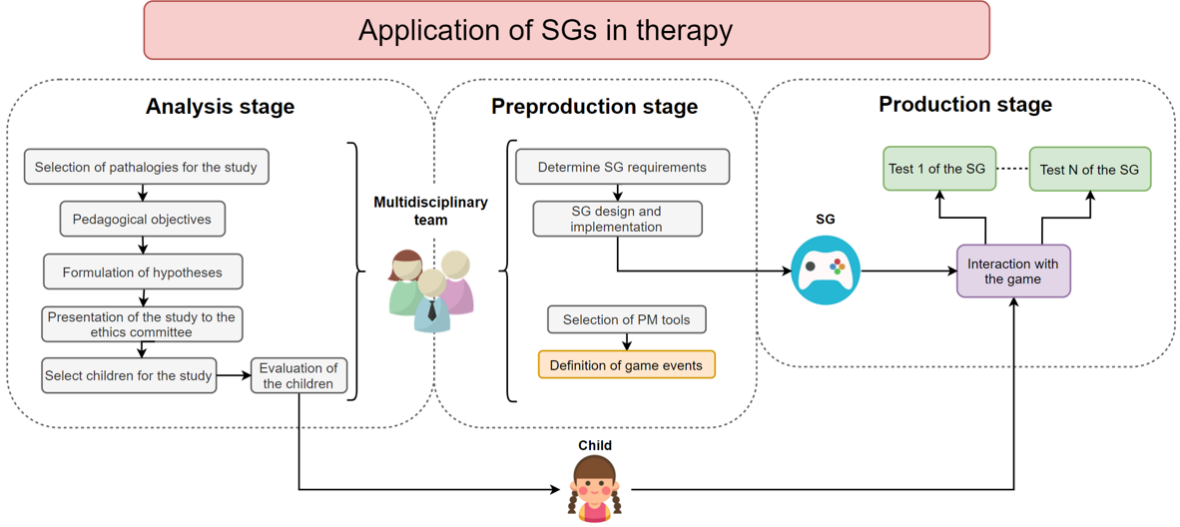
Phase 1: deployment of a screening tool for school-aged children from a health care perspective. PM: process mining; SG: serious game.

In addition, the proposed solution is oriented toward its use among school-age children. As children are the end users for whom the solution is developed, it should take into account not only the characteristics of the disorder to be studied but also the characteristics of the age range of the users. In other words, the game should present an attractive and a motivating environment for children, making them increase their commitment when interacting with the tool. Figure 1 presents an outline of the process of developing the game as a screening tool.

Amalia’s Planet is set, as the name suggests, on a planet consisting of multiple modules (“habitats” or “islands”) of different types. Each “habitat” is home to a different type of animal and incorporates multiple mini-games, all created with a simple cartoon design, making them easily accessible to primary-school children. The animals chosen are those that normally form herds or flocks, and they emit different sounds that are collectively handled as “noise.” This makes it possible to establish noisy backgrounds that produce sound and visual distractions for the child and thus forces them to train listening skills where they have difficulties.

Located in Antarctica, the habitat presented in this paper focuses on the assessment of certain skills affected by various CAPD-related pathologies. More specifically, the skills assessed in the first module are auditory discrimination, sound localization, auditory memory, and auditory separation in noise. For this, the habitat has a series of mini-games, which sequentially assess the mentioned skills. Each of these mini-games provides a series of stimuli and rewards throughout the game so that the player’s motivation is maintained.

**Figure 2:**
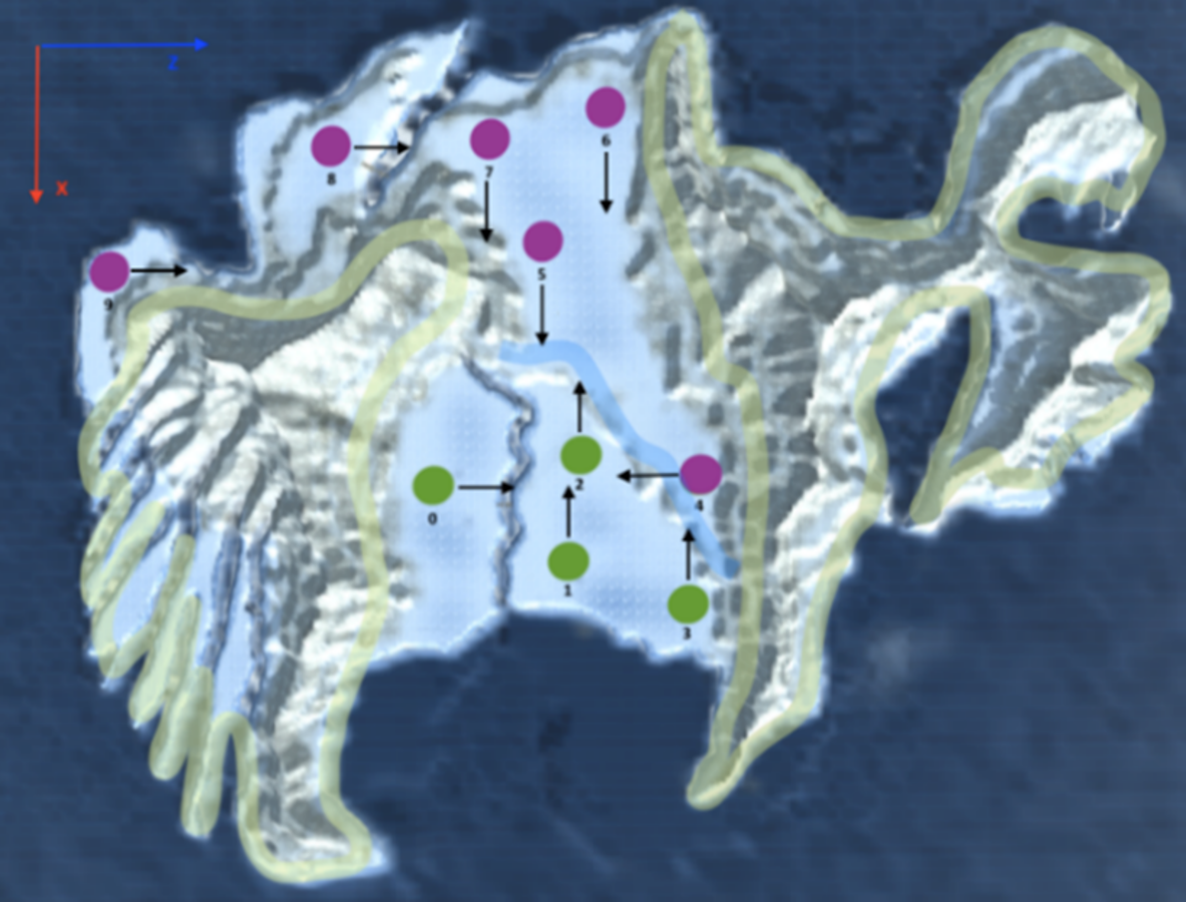
Semirandom distribution of start points and locations of the group of penguins. The yellow lines mark the unreachable areas. The blue line is a slope in the terrain. The green and violet dots are the possible positions of the player and the flock.

As the first mini-game in the Antarctic habitat, the child has to find a stray baby penguin of a herd in a wide area guided only by the sound generated by the animal (auditory localization). The original positions assigned to both the penguin and player are randomly chosen by the game each time the game starts (Figure 2). As a measure of the individual’s performance in this test, the time taken to find the little penguin is collected. Once the child finds the little penguin, this achievement is celebrated by the herd through an applause given to the player.

**Figure 3:**
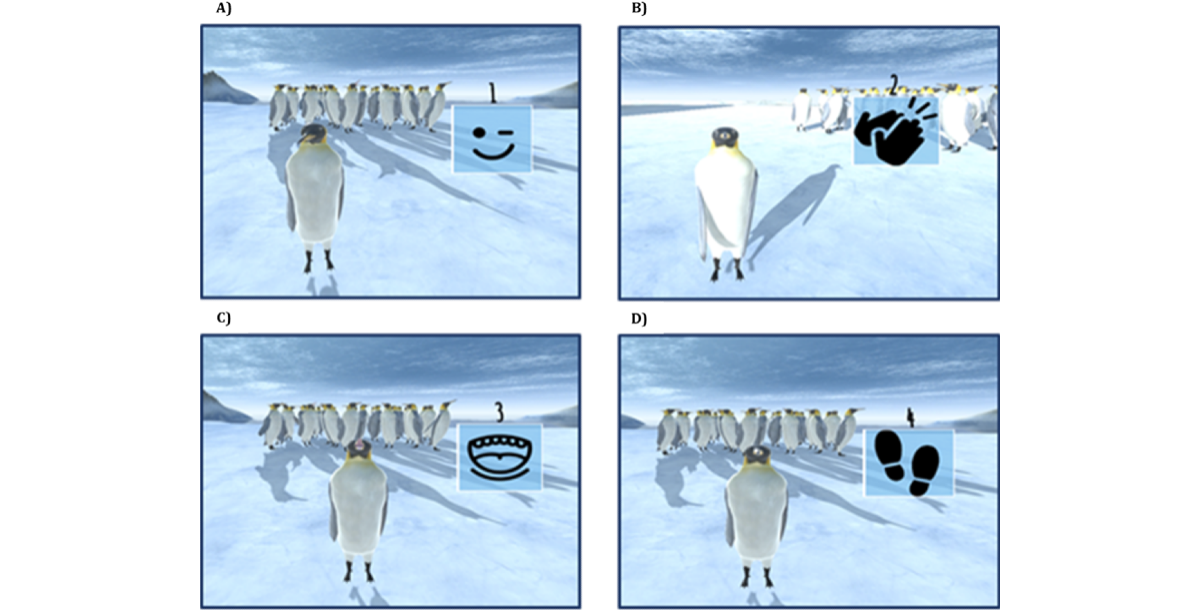
Sequence exposure in the second mini-game.

Once the player passes the first test, the second mini-game starts automatically (Figure 3). This is an auditory memory game. Playing with the head penguin, the child must be able to memorize the sequences of sounds produced by the penguin. Each of the sequences emitted by the head penguin can comprise a series of sounds that the player would have to remember: clapping the wings, twinkling an eye, rattling the beak, and stomping a foot. For the player to become familiar with the aforementioned sounds, the penguin first exposes them together with an animated movement (visual aid). Once the child is deemed to have the necessary information to play the game independently, the penguin hides within its huddle (disappearance of the visual aid) and proceeds to emit sequences of sounds in a given order. The player must remember both the sounds heard and the order in which they were produced to reproduce them once the penguin finishes emitting the sounds.

**Figure 4:**
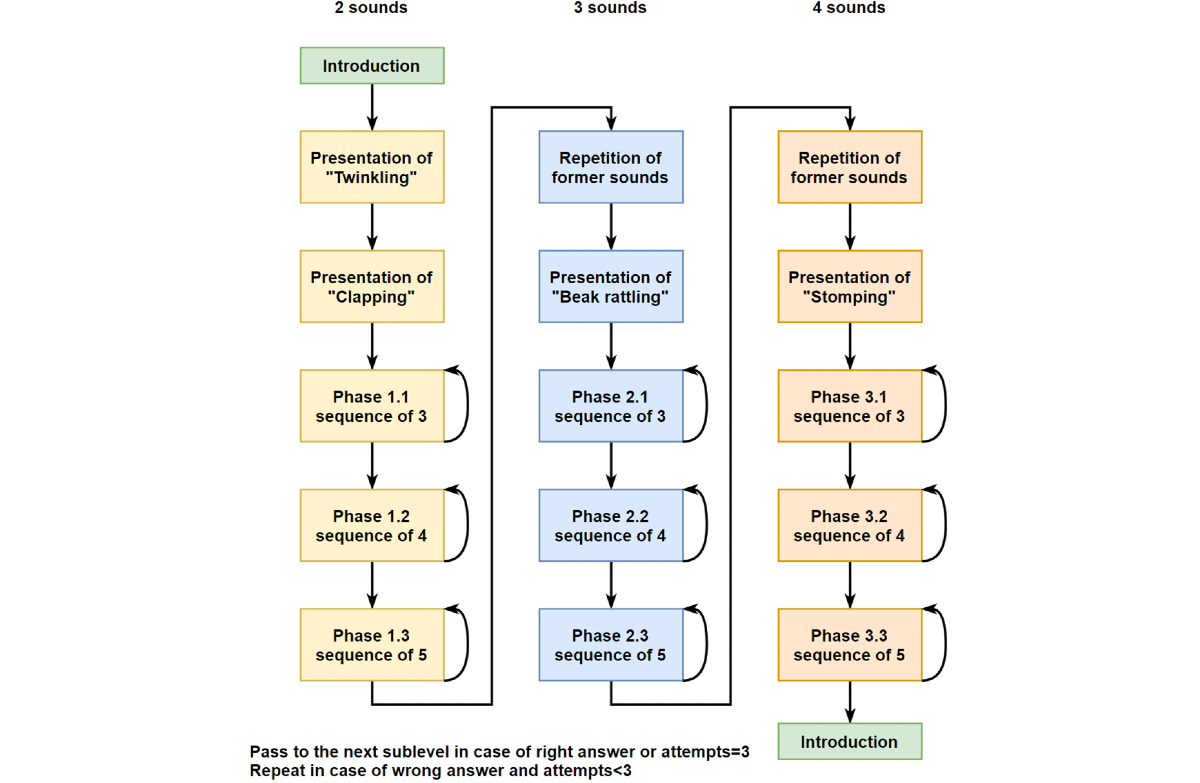
Structure of levels and sublevels to pass in the second mini-game.

With regard to the length of the sequences, they become more complex as the child passes the tests. The number of sounds to be distinguished and the length of the sequence emitted by the animal gradually increase in 3 different levels of difficulty, each of which consists of 3 sublevels (Figure 4). In addition, each sublevel provides the individual with 3 opportunities to pass, and if all 3 opportunities are exhausted without success, the sublevel and its corresponding level shall be considered as not passed. If the player manages to pass any of the levels, applause starts to ring out, and the penguin congratulates the player so that they feel motivated and satisfied with the task performed. If the player does not manage to complete any of the tasks correctly, the penguin encourages them to continue playing (thereby trying to avoid frustration and abandonment) and repeats the sequence of sounds that the child has to recreate.

In addition, the game allows the configuration of this auditory memory test for the evaluation of auditory discrimination skills and sound separation in noise. If desired, the penguin colony hiding the main penguin will emit noise to distract the player, demanding higher auditory abilities. This background sound is adjustable for each player and can vary between 0 dB and 30 dB.

### Amalia’s Planet as Its Self-evaluator

The postproduction stage “corresponds to the evaluation of the serious game, where an evaluation model is proposed, taking into account 2 roles of evaluators, the end user and the expert” [25]. It is at this stage that the previously mentioned PM techniques were introduced as a novel tool for the validation of the proposed solution. However, as reflected in Figure 1, it was at the preproduction stage that the *event logs* that would be analyzed later have to be determined.

Because information was collected in the system logs, it allowed the definition of events by the team, which provided flexibility when addressing problems. Looking at the structure of the game (Figure 4) and with the idea of knowing the behavior of the players throughout the game, the team defined the events according to the result that the player had obtained in each sublevel. At this point, 3 possible outcomes were defined:

1. Passing the sublevel: the player reproduces all the sounds of the sequence in the correct order.
2. Omitting the sublevel: the player does not reproduce all the sounds in the sequence. This result was considered a failure.
3. Failing the sublevel: the player either does not reproduce all the sounds of the sequence correctly or does not reproduce the sounds in the correct order.

**Figure 5:**
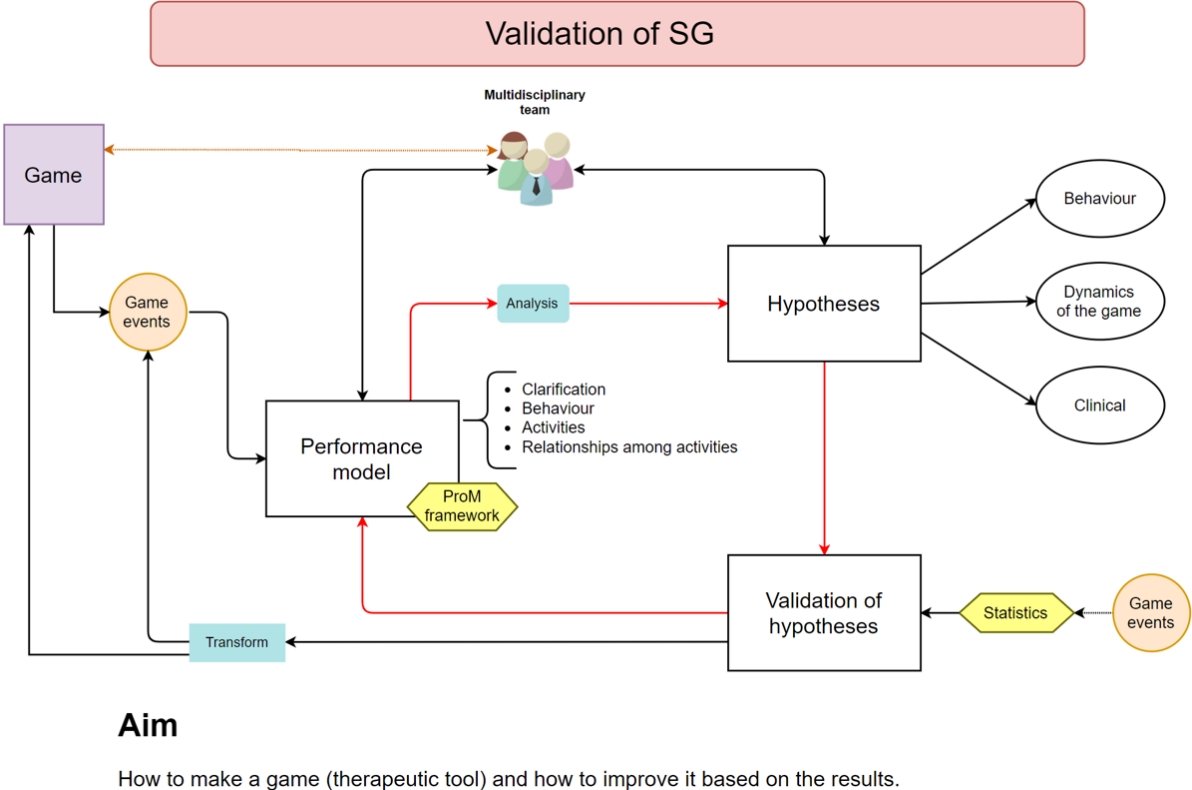
Phase 2: Proposed Amalia’s Planet validation process. SG: serious game.

Thus, the traces of the event logs consisted of events that report the path followed by each player throughout their progress in the game. This definition of events allowed us to know the basic behaviors of the players, such as the number of attempts a player needed to pass a level and the nature of the level (omission or failure). In addition, it allowed us to recognize patterns of interest that could occur, such as more frequent cycles, traces, or paths among players that are not easily perceived using conventional statistical tools. Figure 5 schematically shows the tool validation process proposed by the development team.

The information provided by PM about the system’s functioning, performance, and use as a tool for achieving specific objectives can serve as a source of input for various purposes, which highlights PM’s capacity in this regard. Not only can conclusions be drawn about the achievement of the objectives by users, but hypotheses that could not be validated or were poorly formulated at the outset can also be modified because of the new information obtained. In fact, new hypotheses can be put forward on the basis of behaviors collected in the *event logs*, which had previously not been taken into account because of a lack of knowledge.

As shown in Figure 5, the validation process of the hypotheses raised affects previous steps, such as the definition of the game events or the very conception and design of the game. This iterative and dynamic process is what allows the solution to be fine-tuned. The ultimate goal of this loop process is that, based on the information self-generated by the game, the system itself is nourished, updated, and adapted to the demands and characteristics of the player, as well as to the process of analysis of this information presented to the team of experts.

### Experiment and Hypothesis

Amalia’s Planet was tested twice in a school in the Community of Madrid, Spain.

Information was collected through interviews with parents about their children’s medical history related to previous hearing pathologies, such as previous hearing infections (otitis, drains, and secretions) and ear surgeries, and whether their children had a diagnosis of attention-deficit/hyperactivity disorder (ADHD) or learning difficulties [32,33]. Hereafter, this group of conditions will be referred to as previous hearing pathologies.

To evaluate the achievement of the discriminating objective of the game (screening tool), the interdisciplinary team proposed the following hypotheses:

1. There is a difference in gameplay performance between children with previous hearing pathologies and children without previous hearing pathologies.
2. “Healthy” players learn faster. Children who have not had any of the aforementioned conditions are expected to improve their gameplay performance faster than children with a diagnosed pathology.
3. Children learn while they play. Regardless of whether children have had a hearing condition, it is interesting to know whether the game improves their trained skills.

To test the formulated hypotheses, a statistical study of the difference in means between the 2 groups of children (with and without previous hearing difficulties) was conducted. In addition, different scatter plots were studied with respect to measures of central tendency with the aim of finding atypical behaviors that reveal the presence of individuals at risk. These hypotheses are assessed later in this paper.

## Results

### Sample Summary

The sample size was determined in such a way that, according to the central limit theorem, the statistical distribution of each of the populations considered for the study (children with previous hearing pathologies, children without previous hearing pathologies, distribution of children by sex, etc) was correctly reflected in the collected data [34]. Thus, theoretically, a sample size of 50 children was determined for each sample group (n>30), with a total sample of 100 children in the study. Nevertheless, owing to the limited availability of users of interest for the study and some problems in data collection using the tool (3 players were excluded), the final size of the sample used for this study was 87 children.

The sample consisted of children of both sexes (50 female and 37 male children) aged between 5 and 14 (mean 8.87, SD 1.74; median 9) years. Most of the players (76/87, 87%) were aged between 7 and 11 years, of whom 55% (42/76) were female and 45% (34/76) were male, maintaining approximately the initial distribution of the sample.

Regarding the pathology line, without taking into account sex or age, 45% (39/87) of the players had or used to have at least 1 pathology among those contemplated in the study, resulting in 55% (48/87) of “healthy” participants. The sex ratio was as follows: 57% (50/87) female and 43% (37/87) male. Health information obtained through the parent questionnaire revealed the following classification by previously identified pathology: (1) 55% (48/87) of the sample had a history of otitis, of whom 33% (16/48) had major complications, although only 28% (24/87) had undergone a surgery for drainage; (2) 3% (3/87) were diagnosed with ADHD; and (3) a similar percentage 3% (3/87) had learning disorders.

The sample included children with a history of hearing pathologies and children without a history of hearing pathologies. Hence, focusing on the players with some previous pathology (39/87, 45%), there were 49% (19/39) of players with just 1 pathology, 31% (12/39) of players with 2 different pathologies, and 21% (8/39) of players with up to 3 types of pathologies. Overall, 92% (36/39) of the players with at least 1 pathology had or used to have otitis.

Among the 19 children with just 1 affliction, 84% (n=16) had otitis (a percentage close to the prevalence of otitis in the total population of 87 children, which is estimated at 80% [35]), and 11% (n=2) had ADHD, making it the second most frequent pathology appearing individually. Generally, suppurations and learning disorders appeared to be accompanied by at least 1 other type of pathology in all cases. The most common combination of afflictions was made up of otitis and suppurations, which was present in 11 children (55% of the players with at least 2 pathologies). In terms of sex, the percentage of female players with some kind of affliction was close to the percentage of male players with some kind of affliction: of the 50 female players, 22 (44%) had a pathology, and of the 37 male players, 17 (46%) had a pathology.

### Overview of the Obtained Health Results

#### Results Obtained in Test 1

For this first test, a sample of 59 children was available. Among the 59 children, 29 (49%) had previous hearing pathologies, with a total of 14 (48%) male and 15 (52%) female children. On the other hand, a total of 51% (30/59) children were part of the group of children who were not diagnosed with hearing pathologies, with a total of 50% (15/30) male and 50% (15/30) female children.

The percentage of players with afflictions who managed to finish the game successfully was lower than the percentage of “healthy” players (11/29, 38% of players with some affliction against 16/30, 53% of “healthy” players). To determine whether this difference in the level of playing between the different groups was statistically significant, a chi-square test was performed, obtaining a *P* value of .24. Therefore, whether the child had or used to have a hearing pathology did not seem to affect their ability to successfully complete the game. However, it was interesting for the team to answer the following question: is it possible that some of the children initially classified as “healthy” players may in fact have one of the pathologies considered in the study or a different one and have not been diagnosed? Taking the following variables as references, throughout the collection of the information, it was observed that there were certain players initially cataloged as “healthy” whose level of gaming stood out compared with the level of playing of their peers:

- TOTAL_ATTEMPTS: the number of attempts the player has made throughout their game
- TOTAL_ACCURACIES: the number of successes the player has produced throughout the game
- FAILURE_TOTAL: the total number of failures the player has faced during the game
- TOTAL_SUBS: the number of sounds omitted by the player throughout the game

**Figure 6:**
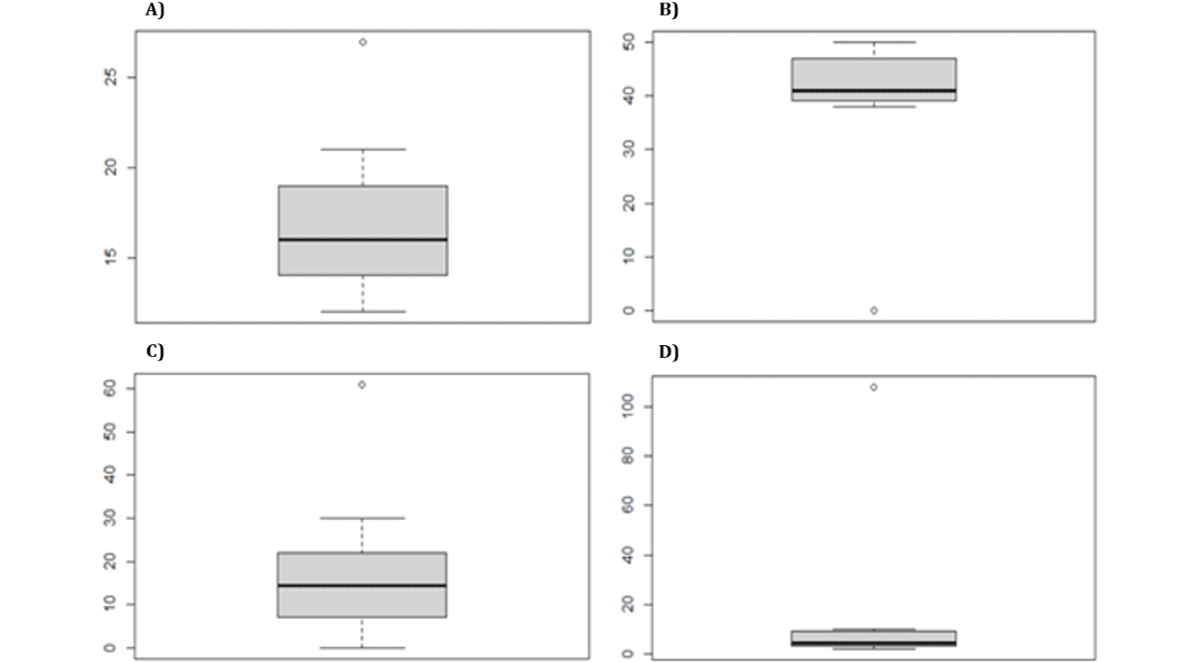
Test 1: (A) total number of attempts for healthy players who did not complete the game, (B) total accuracies for healthy players who did not complete the game, (C) total number of failures for healthy players who did not complete the game, and (D) total number of omissions for healthy players who did not complete the game.

Figures 6A to 6D show (from left to right) the existence of players initially classified as “healthy” who, compared with their group, showed atypical behaviors in their results, particularly, the players who did not manage to pass the game, presenting a large number of errors or omissions throughout their game (ID 30 and ID 74).

#### Results Obtained in Test 2

For the second test, a sample of 70 children was available. Among the 70 children, 28 (40%) had previous hearing pathologies, with a total of 13 (46%) male and 15 (54%) female children. On the other hand, a total of 60% (42/70) children were part of the group of children who were not diagnosed with hearing pathologies, with a total of 45% (19/42) male and 55% (21/42) female children.

In this second test, most players managed to pass all the levels of the game. There was a clear difference between the percentage of children with previous pathologies who managed to finish the game completely and the percentage of children without previous pathologies who managed to finish the game completely (26/42, 62% of the players without pathology against 12/28, 40% of the players with some type of diagnosed pathology). To determine whether this difference was statistically significant, a chi-square test was performed, obtaining a *P* value of .19. Therefore, at a confidence level of 80%, there was no statistical evidence to reject the hypothesis that having the afflictions in question affects the ability to complete the game successfully.

**Figure 7:**
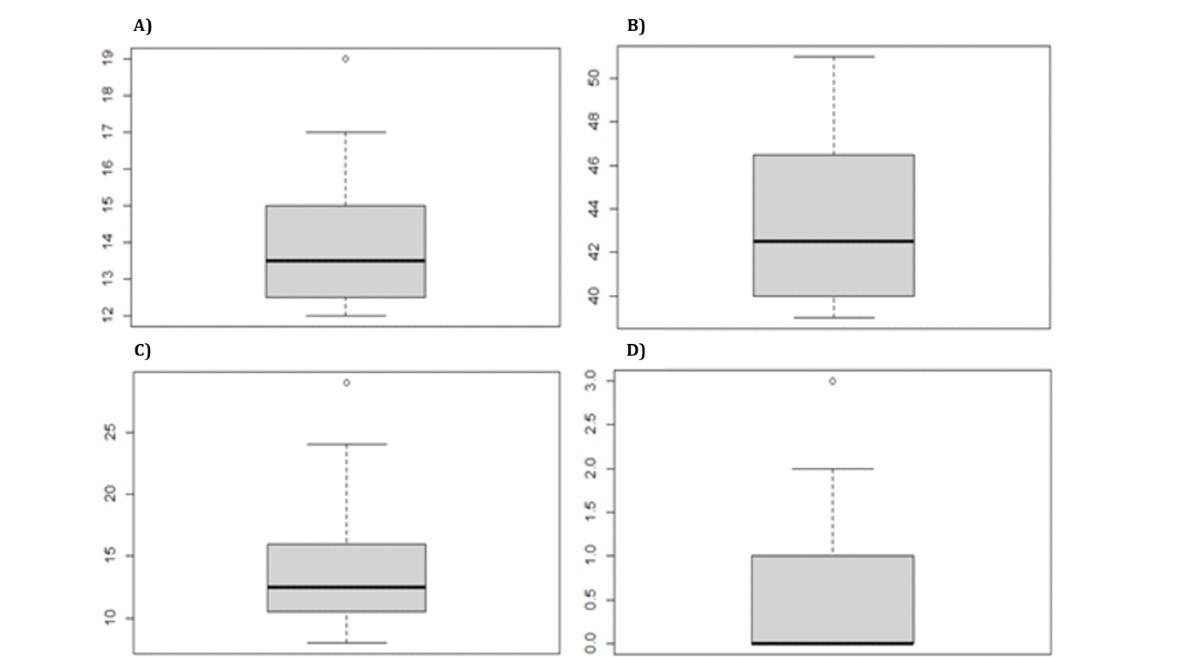
Test 2: (A) total number of attempts for healthy players who did not complete the game, (B) total accuracies for “healthy” players who did not complete the game, (C) total number of failures for healthy players who did not complete the game, and (D) total number of omissions for healthy players who did not complete the game.

To determine whether the players who stood out in test 1 maintained the trend in the second test, the variables mentioned earlier were analyzed again to study their distribution in the sample. Figure 7 shows that these players maintained the same tendencies along the second trial.

### Overview of the Obtained Game Validation Results

As noted earlier, the achievement of the test objectives should not be the only measure of the test’s fitness for purpose, but aspects such as the time required to pass each of the tasks and the different event traces constituted by each player can be sources of information to be considered. To this end, the team used the ProM (Prom Software Inc) software, which is an extensible framework that supports a wide variety of PM techniques in the form of plug-ins [27]. By evaluating the different event logs defined in the previous stages, the abovementioned features can be evaluated and, if necessary, modified further by adapting the tool to its final objective.

**Figure 8:**
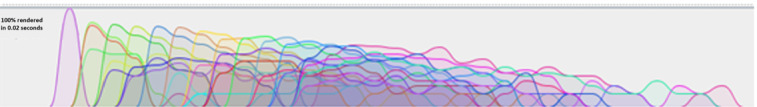
Duration of events.

Figure 8 depicts the duration of each of the events that were defined in the initial stage. Based on the understanding that the duration of an event is the time that elapses from the time when the “faster” player initializes it until the time when the “slower” player finishes it, this graph can reveal the existence of events that pose a greater challenge to the individual. To avoid the feeling of frustration that the player may experience, and the consequent decrease in performance, this particular feature of PM techniques allows the team to modify such game events.

**Figure 9:**
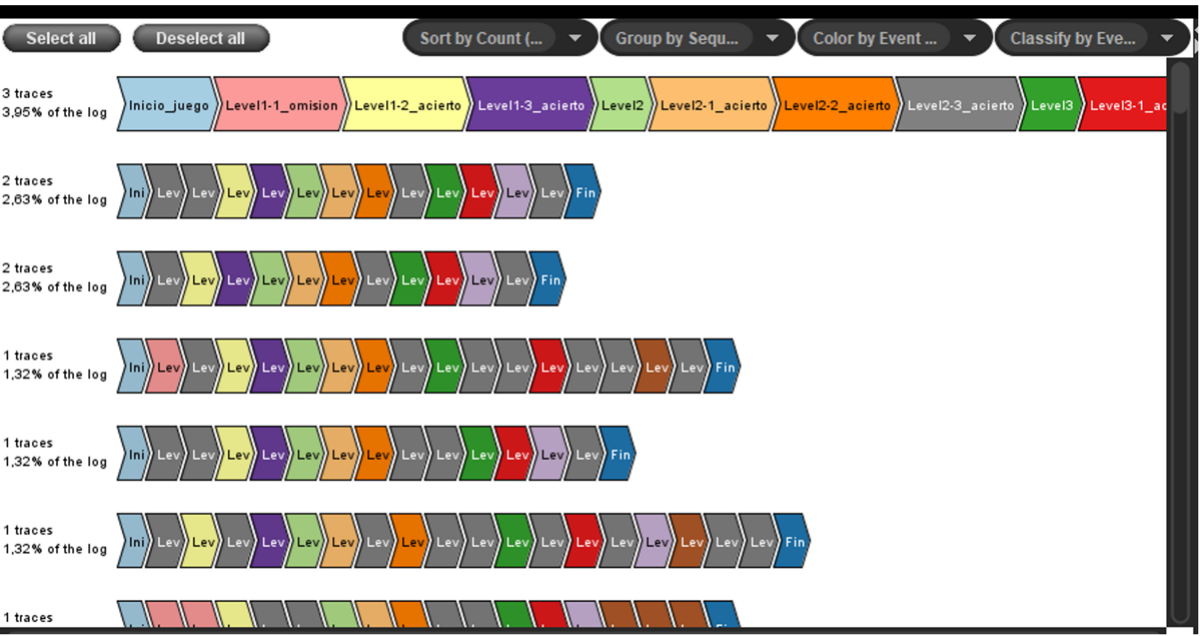
Obtained traces and their frequency.

Furthermore, the study of event traces and the frequency with which they appear in the collected sample serves as a mirror of the correct functioning of the game (Figure 9). Thus, it is possible to detect structural faults in the tool, such as jumps between levels that are not consecutive, backtracking that is not allowed, and advancing to a new level without having completed the previous one. All these constituent faults can be corrected by the technical part of the development team, ensuring the correct functioning of the tool in future tests.

**Figure 10:**
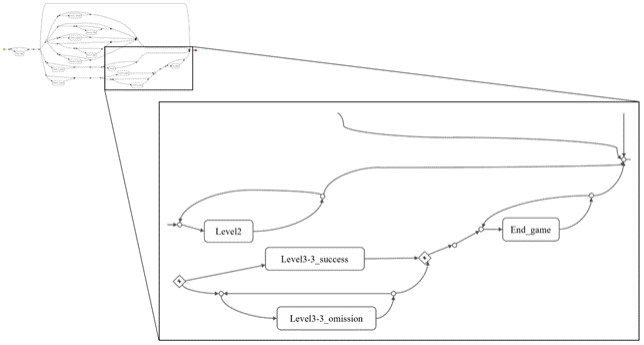
Business Process Model Notation with inductive visual miner.

Finally, another graph of interest that can be obtained using the ProM software is the so-called Business Process Model Notation [27]. This graph provides information about the route that different players followed to pass the game, identifying the main events that each player went through. Furthermore, loops and cycles can be detected using this graph; thus, the team can deduce the possible learning resources of the players (Figure 10).

## Discussion

### Principal Findings

SGs are increasingly being used as support tools for the development and improvement of skills in individuals of different age ranges in areas such as education and health. In addition, it is nowadays demonstrated that an early diagnosis of CAPD in an individual is a desirable characteristic from the point of view of the consequent therapeutic process [36]. In other words, school-age children who are screened for CAPD may benefit greatly from the treatment of CAPD symptoms. Despite this, it is currently not possible to find a tool that brings together these 2 ideologies to minimize, or even eliminate, the afflictions that this disorder causes in those who have it.

On the basis of the conception and development of an SG, Amalia’s Planet, and its use in a real school environment, the research team was able to contrast the different hypotheses put forward in previous sections regarding the suitability of the use of SGs as screening tools for CAPD. More specifically, this study showed that once the users become familiar with the game and its rules, users who were affected by auditory pathologies related to the disorder in question perform less well on the tasks they are given. More specifically, at a confidence level of 80%, there was no statistical evidence to reject the hypothesis that there is a difference in gameplay performance between children with hearing pathologies and children without hearing pathologies. That is, the fact that the player had any of the pathologies considered in this study directly affects their performance in the game. Moreover, the abovementioned statement only supports the hypothesis that “healthy” children learn faster than children with a diagnosis. Finally, an improvement in the performance of the players could be observed when comparing their performance in test 1 with that in test 2.

One of the main results obtained in this study is that using the proposed solution, the team was able to detect the presence of 2 users who were initially assigned to the group of “healthy” individuals owing to the lack of diagnosis but who presented a gameplay performance more similar to that of the group of at-risk individuals. Such an outcome allows family members, educators, and health professionals to pay more acute attention to these users. Such children can then be examined to determine whether their performance was altered by the presence of an auditory processing pathology or by other external factors.

At this point, it is worth highlighting the fact that if, using traditional statistical techniques on extreme values, both users were eliminated from the sample, and the data were reanalyzed, the results obtained would have been even more discriminant. For both tests 1 and 2, with statistical confidence levels of 85% and 95%, respectively, there would have been no statistical evidence to reject the hypothesis about the difference in performance between “healthy” players and those who had been diagnosed.

In turn, the use of PM techniques as a tool for evaluating the suitability of the solution to the problem posed allowed the team to develop different methods for improving the game. In particular, by studying the duration of the game events, the team detected some phases of the game that can generate frustration in the players, thus reducing their level of attention and, consequently, their performance. In addition, different learning resources used by the players were detected. By studying cycles and loops along the player’s traces, learning resources based on failure and omission that were used by the player to subsequently complete the levels could be detected. Finally, the team was able to detect jumps between nonconsecutive levels of the game. All these features to be improved in the tool could be addressed in future versions, thanks to the validation of the proposed solution through PM techniques.

### Limitations of the Study and Future Works

After testing the developed tool, the team was able to detect a series of aspects to improve in the application. First, at the time of data analysis, a lack of information was detected from a total of 3 users. This lack of information due to an error in the deployment of the solution led to the exclusion of these players from the final analysis. Moreover, in the first game in which the child has to find the baby penguin, a bug was detected. Here, some players had to cross a bridge to reach their goal. However, some of them fell from the bridge into the water and required the help of adults to continue, adding time to their markers and lowering their score. Moreover, thanks to the use of PM tools, events in which the dynamic followed in the game was not established could be detected a priori. Thus, in Figure 10, a jump from the initial event to the final event without going through any intermediate states can be detected. In addition, in other cases, player games were detected in which the game “jumps” from one level to another, ignoring intermediate sublevels.

Finally, events that were “excessively” long compared with others were detected. These events will have to be reviewed, as they can suppose a defective performance on the part of the tool or a high level of complexity for the players involved.

In addition to the revision and correction of the mentioned errors, the team of authors is currently developing new “habitats” for the Amalia’s Planet ecosystem, which covers new aspects related to CAPD screening and were not included in this first version.

### Conclusions

CAPD is a disorder that is difficult to detect during childhood, and its early detection is a challenge to be solved in the near future using quick and easy-to-apply screening tools. To our knowledge, this is the first study that unifies the ideology of a screening test for CAPD in school-age children and the use of SGs to support the diagnosis and consequent treatment of CAPD. Despite the need for further testing of the usability of the tool and the improvement of certain functional aspects of the tool, the development team was able to obtain results with statistical evidence that support the use of Amalia’s Planet as a screening tool for children at risk for CAPD. Amalia’s Planet can be expanded to include games that assess other forms of afflictions experienced by children with the disorder under study.

## Abbreviations

ADHD: attention-deficit/hyperactivity disorder
CAPD: central auditory processing disorder
MECONESIS: metodología para la concepción de juegos serios para niños con discapacidad auditiva
PM: process mining
SG: serious game
